# Micropatterning Decellularized ECM as a Bioactive Surface to Guide Cell Alignment, Proliferation, and Migration

**DOI:** 10.3390/bioengineering7030102

**Published:** 2020-08-31

**Authors:** Emily Cady, Jacob A. Orkwis, Rachel Weaver, Lia Conlin, Nicolas N. Madigan, Greg M. Harris

**Affiliations:** 1 Department of Chemical and Environmental Engineering, University of Cincinnati, Cincinnati, OH 45221, USA; cadyea@ucmail.uc.edu (E.C.); orkwisja@ucmail.uc.edu (J.A.O.); weaverrv@ucmail.uc.edu (R.W.); conlinlg@ucmail.uc.edu (L.C.); 2 Department of Neurology, Mayo Clinic, Rochester, MN 55905, USA; Madigan.Nicolas@mayo.edu; 3 Department of Biomedical Engineering, University of Cincinnati, Cincinnati, OH 45221, USA; 4 Neuroscience Graduate Program, University of Cincinnati College of Medicine, Cincinnati, OH 45267, USA

**Keywords:** extracellular matrix, decellularization, micropatterning, cell alignment, biomaterials, tissue engineering

## Abstract

Bioactive surfaces and materials have displayed great potential in a variety of tissue engineering applications but often struggle to completely emulate complex bodily systems. The extracellular matrix (ECM) is a crucial, bioactive component in all tissues and has recently been identified as a potential solution to be utilized in combination with biomaterials. In tissue engineering, the ECM can be utilized in a variety of applications by employing the biochemical and biomechanical cues that are crucial to regenerative processes. However, viable solutions for maintaining the dimensionality, spatial orientation, and protein composition of a naturally cell-secreted ECM remain challenging in tissue engineering. Therefore, this work used soft lithography to create micropatterned polydimethylsiloxane (PDMS) substrates of a three-dimensional nature to control cell adhesion and alignment. Cells aligned on the micropatterned PDMS, secreted and assembled an ECM, and were decellularized to produce an aligned matrix biomaterial. The cells seeded onto the decellularized, patterned ECM showed a high degree of alignment and migration along the patterns compared to controls. This work begins to lay the groundwork for elucidating the immense potential of a natural, cell-secreted ECM for directing cell function and offers further guidance for the incorporation of natural, bioactive components for emerging tissue engineering technologies.

## 1. Introduction

A significant portion of tissue engineering research focuses on the ability to derive native biological structures as biomaterials for regenerative purposes [1,2,3,4,5,6,7,8]. Biomimetic structures, in particular, strive to recreate naturally regenerative microenvironments to promote desired cell behavior and mitigate inflammatory responses [9]. Recently, the extracellular matrix (ECM) has been targeted as a bioactive substrate for tissue repair [10,11,12,13]. A naturally cell-assembled ECM is able to provide physical support to tissue while concurrently mediating cell and tissue behavior [14,15]. Therefore, desired phenotypic outcomes in tissue engineering such as cell alignment, proliferation, and differentiation are inherently intertwined with the ECM structure and composition [16]. As such, tissue regeneration is largely dependent on the biomechanical and biochemical structure and components of the ECM.

In order to translate solutions using ECM to the next level, the ability to engineer and guide specific orientations while maintaining the native matrix components is necessary. While individual ECM components vary greatly between tissues, glycosaminoglycans (GAGs) and glycoproteins, including collagen and fibronectin (FN), are generally ubiquitous [14,16,17]. To create the ECM, the secretion and organization of the macromolecules are mediated by cells themselves [18]. For FN in particular, as dimers are secreted, they are assembled into a 3D ECM by cellular tension and capable of binding other components [11,13,15,18]. The cell-secreted ECM then has the potential to be decellularized to remove cells, leaving only a cell-assembled decellularized ECM (dECM). Decellularization assays generally use a series of wash and lysis buffers to remove cells with minimal degradation of the ECM [19]. The dECM has high potential to be used as a bioactive surface due to its natural composition and organization of macromolecules, with the additional advantage of growth factor sequestration. Regarding this, researchers have demonstrated the ability of dECM to influence cell proliferation, migration, matrix remodeling, and other cellular responses [11,18,20,21,22].

However, ECM organization and alignment are imperative to the functionality of tissue throughout the body, and a decellularized matrix generally lacks the alignment of native ECM, leaving a critical gap for tissue engineering strategies [23]. For example, skin possesses an organized, aligned ECM, composed primarily of collagen, which contributes to the unique tensile strength and elasticity of the organ [24]. The alignment of the proteins making up the ECM can also be strongly dependent on the alignment of cells [25,26]. It is well established that alterations in the chemical and structural characteristics of cell culture surfaces can impact cell behavior, including alignment [27,28,29,30,31]. Likewise, topographically patterned cell-culture surfaces can guide and organize cells and secreted ECM components [26,28,32]. Furthermore, while a variety of micro- and nanopatterned biomaterials have been generated for cell alignment, the fabrication of easily micropatterned substrates using strictly physical cues to align cells, and therefore ECM, holds potential for biomaterials for tissue engineering.

To address the need for patterned, aligned ECM, this work fabricated micropatterned polydimethylsiloxane (PDMS) substrates to control the alignment of cells and the resulting dECM, thereby replicating the alignment found within native tissue. With the importance and function of ECM in all tissues already well established, the optimization of substrate topography for the organization of a cell-derived ECM has not been established. The guided assembly of a cell-derived ECM has potential for the controlled adhesion, alignment, and migration of cells for use in various tissue regeneration and biomedical applications [33]. Following analysis of the viability of micropatterns with differing dimensional geometry on ECM alignment, the resulting matrices were analyzed for their impact on cell behavior. For tissue with a defined spatial orientation such as skin, peripheral nerve, and bone, this work introduces a novel approach for creating bioactive substrates for use as regenerative biomaterials with a native tissue structure and composition.

## 2. Experimental Procedures

### 2.1. Micropatterning Polydimethyl Siloxane Substrates

Micropatterned surfaces were fabricated according to previously published soft lithography protocols [30,34]. Briefly, lined patterns with various dimensions were designed using Autodesk AutoCAD, and high-resolution quartz photomasks were produced (Frontrange Photomask). A cleaned silicon wafer was spin coated using SU-8 2010 photoresist (MicroChem, Newton, MA) to achieve 10 μm height, following the parameters in the MicroChem SU-8 Photoresist data sheet. The wafer was then soft baked at 95 °C for 3 min. Patterns were generated on the photoresist-coated silicon wafer with a photomask using a Solitec 3000IR Mask Aligner and exposure to 130 kJ/cm^2^. The surface was post baked at 95 °C for 4 min and rinsed using MicroChem’s SU-8 developer with light agitation. After a final rinse with developer, the surface was sprayed or washed with isopropyl alcohol followed by air drying. For a 5 μm pattern height, SU-8 2005 was spin coated at 3000 rpm, soft baked for 2 min, exposed at 105 mJ/cm^2^, and post-baked for 3 min.

Sylgard 184 polydimethylsiloxane (PDMS)(Dow Corning) base and curing agent were mixed at a 10:1 ratio, mixed well, and poured over the patterned wafer. PDMS was set overnight at 37 degrees Celsius to dry. PDMS substrates were cut out from the silicon master, and the patterned dimensions were verified using microscopic imaging to quantify the groove width, ridge width, and depth. Following initial confirmation, patterns were analyzed every 15 substrates to maintain consistency.

### 2.2. Substrate Preparation and Cell Culture

To prepare PDMS for cell culture, substrates were washed in 70% ethanol, dried with filtered air, and exposed to UV light for 7 min. Surfaces were moved into a sterile cell culture hood and placed at the bottom of 12- or 24- well cell culture plates on a coverslip. Substrates were then rinsed with 70% ethanol and washed twice with sterile 1× DPBS (Gibco^®^, Grand Island, NY, USA). NIH 3T3 full growth medium (Hyclone™ Dulbecco’s High Glucose Modified Eagle Medium (DMEM/High Glucose), 10% Bovine Calf Serum (BCS), and 1% penicillin–streptomycin were added to each well, covering the cell culture surface. The plate was then incubated for approximately 30 min prior to cell seeding.

NIH3T3 fibroblast cells (ATCC CRL-1658™, Manassas, VA, USA) were seeded at approximately 70,000 cells/cm^2^ and maintained at 37 degrees Celsius, 90% relative humidity, and 5% CO_2_. Culture medium was replaced after 12 h with fresh full growth medium containing 100 µg/mL ascorbic acid. After each additional 48 h of culture, the culture medium was changed to that containing 50 µg/mL of ascorbic acid. The cells were cultured for 2 days past confluency (approximately 7 days in total).

### 2.3. Preparation of Decellularized ECM and Culture

Substrates were decellularized as previously described [11,13]. In brief, the substrates were washed twice with 1× PBS and rinsed twice with wash buffer solution containing 0.1 M sodium phosphate (Na_2_HPO_4_), 2 mM magnesium chloride (MgCl_2_), and 2 mM Egtazic acid (EGTA), adjusted to a pH of 9.6. Then, the surfaces were incubated in lysis buffer containing 8 mM Na_2_HPO_4_ and 1% NP-40 for 15 min at 37 °C. The lysis buffer was removed, fresh lysis buffer was added, and the substrates were incubated for 15 min at 37 °C. Finally, the lysis buffer was removed, fresh lysis buffer was added, and incubation was performed for 1 h at 37 °C. The lysis buffer was removed, and the surfaces were rinsed twice with a second wash buffer containing 10 mM Na_2_HPO_4_ and 0.3 M potassium chloride. Each substrate was then rinsed four times with DI water. The surfaces were stored in 1× PBS at 4 °C for at least 3 days before analysis or further culture. For reseeding cells onto decellularized matrices, PBS was removed gently from the substrates as to not disrupt the ECM, and the substrates were seeded at approximately 50,000 cells/cm^2^.

### 2.4. Immunofluorescence and Microscopy

Following culture, cells were fixed with a 4% formaldehyde solution for 15 min at room temperature. Cells that had been stained for cell cytoskeleton and nuclei were lysed with 0.2% Triton-X solution, incubated for 5 min at 4 degrees Celsius, and stained with rhodamine phalloidin (1:100) in 2% (*w*/*v*) bovine serum albumin (BSA) solution for 30 min at 37 degrees Celsius. To visualize the ECM, R457 rabbit anti-fibronectin polyclonal antiserum (1:100, kindly donated by Dr. Jean Schwarzbauer) [11], followed by Alexa Fluor 488 goat anti-rabbit (1:600), was used. The PDMS surfaces were mounted on slides using approximately 20 μL of ProLong Diamond Antifade Mountant with DAPI and sealed. The samples were imaged using a Nikon Eclipse Ti2 Inverted Microscope with a Nikon DS-Qi2 camera using phase or fluorescence filters and analyzed with the Nikon NiS-Elements: Advanced Research 5.02 software. Cell migration assays were performed using a Tokai Hit Stage Top Incubator system (Model WSKM).

### 2.5. Quantification and Analysis

Cell and ECM alignment quantification was performed using the ImageJ (National Institutes of Health) software. Fast Fourier transform (FFT) Analysis was performed on each individual image cropped to 1024 × 1024 pixels according to established protocols [17,35]. FFT data were then utilized to calculate the full width half maximum (FWHM) for each curve, plotted, and compared across samples. A smaller FWHM represented a tighter intensity peak or a greater frequency of aligned pixels, indicating an alignment of cells or ECM fibrils. Five images from each unique experiment were averaged to quantify the degree of alignment.

### 2.6. Statistics

A two-factor ANOVA was performed to determine the significance of differences between patterned and unpatterned surfaces for each unique experiment. If significance was verified by ANOVA, a Tukey’s two sample *t*-test assuming unequal variance was used for statistical analysis between the PDMS micropatterns and the control, as well as between all possible combinations of micropatterns (50/50 vs. 40/40, 40/40 vs. 30/30, 50/50 vs. 20/50, etc.). All the data are reported as * *p* ≤ 0.05, ** *p* ≤ 0.005, and *** *p* ≤ 0.0005. All the quantified results are presented as the mean ± standard error. Statistics were performed using Origin 9.1 or Excel.

## 3. Results 

### 3.1. Patterning Micron-Level Physical Constraints Guides Fibroblast Adhesion, Alignment, and Proliferation

**Figure 1:**
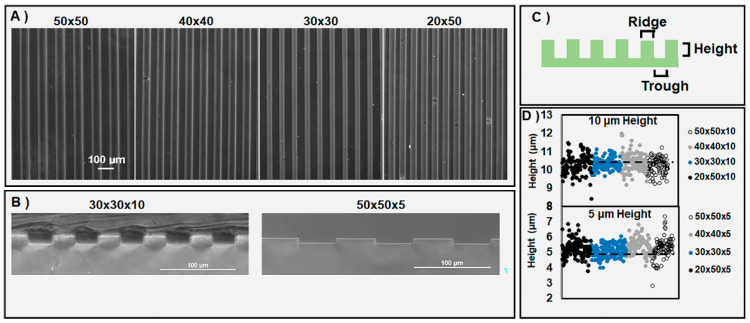
Micropatterned polydimethylsiloxane (PDMS) fabricated via soft lithography. (**A**) Top-down, microscopic view of ridge-by-trough geometry of substrates. Scale bar = 100 µm. (**B**) Cross-sectional representative images show the 3D characteristics of a 30 × 30 pattern with 10 µm height (30 × 30 × 10) and 50 × 50 pattern with 5 µm height (50 × 50 × 5), respectively. (**C**) Three-dimensional PDMS surfaces consisted of three unique dimensions: ridge, trough, and height. (**D**) Pattern heights for four unique dimensions, where dashed lines represent the average heights of all the patterns; n = 5 unique substrates per dimension, with 20 height measurements per substrate.

Three-dimensional PDMS surfaces with micron-scale patterning were developed to determine the effect of pattern geometry on cell adhesion, spreading, and alignment. Soft lithography was utilized to fabricate 3D “grooved” micropatterned PDMS of variable dimensions (Figure 1A and Figure S1). Micropatterns were constructed with specified groove widths, ridge widths, and heights (Figure 1B,C). The micropattern dimensions tested (labeled as ridge by trough dimension) were 50 by 50 μm (50 × 50), 40 by 40 μm (40 × 40), 30 by 30 μm (30 × 30), and 20 by 50 μm (20 × 50), where each pattern was produced at a height of 5 or 10 μm. The patterned PDMS heights were verified through the cross-sectional imaging of the 3D PDMS surfaces, showing the observed average height for the 10 and 5 μm patterns to be 10.33 ± 0.01 and 5.29 ± 0.02 μm, respectively (Figure 1D). The ridge and trough dimensions were analyzed by phase microscopy (Table S1). 

**Figure 2:**
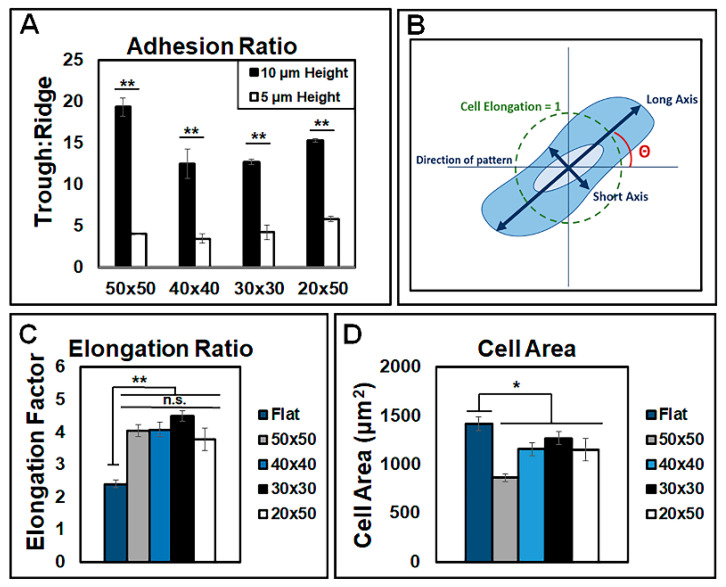
Cell morphology was controlled by micropatterned PDMS surfaces. (**A**) Cell adhesion as ratios of the micropatterning trough and ridge dimensions, for the cells present in the images, for both 10 and 5 µm heights; n = 3 substrates per condition and five images per substrate. (**B**) Visual representation of the quantification method for defining the long and short axis dimensions. (**C**) Cell elongation ratio on micropatterned substrates and (**D**) total area of individual cells cultured on 10 µm-height patterns. n = 4 unique images per condition, with at least 20 cell measurements per image. Data are presented as mean ± SE, where * *p* ≤ 0.05, ** *p* ≤ 0.005.

NIH 3T3 fibroblast cells were cultured on patterned PDMS substrates and analyzed for adhesion, spreading, and alignment to quantify impact of patterning cells three-dimensionally at the micron scale. Upon quantifying the adhesion location of the cells, the cell adhesion ratios showed that the cells were significantly more likely to adhere to the troughs than the ridges of the micropatterns. After 24 h of culture, the cells seeded on 10 μm-height patterns were over 10 times as likely to adhere to the trough compared to the ridge, while the cells seeded on 5 μm-height patterns were approximately five times as likely to adhere to the trough than the ridge (Figure 2A). Initially, cells adhered to the PDMS substrate and elongated with a long axis parallel to the grooved micropattern, whereas unpatterned PDMS showed no specific alignment (Figure 2B,C). Following 24 h of culture, the single-cell morphology on the micropatterned PDMS surfaces was significantly more elongated compared to that of the cells cultured on unpatterned PDMS (Figure 2C). Comparisons across the micropattern dimensions showed no significant difference in cell elongation. The average cell area was then analyzed, with the cells cultured on unpatterned PDMS having an average cell area of 1420.6 μm^2^ at 24 h, while the cells cultured on the 50 × 50, 40 × 40, 30 × 30, and 20 × 50 patterned surfaces with 10 μm pattern heights exhibited spreading areas of 864.3, 1157.2, 1270.6, and 1150.7 μm^2^, respectively (Figure 2D). Overall, the micropatterned surfaces induced significantly lower cell spreading areas.

**Figure 3:**
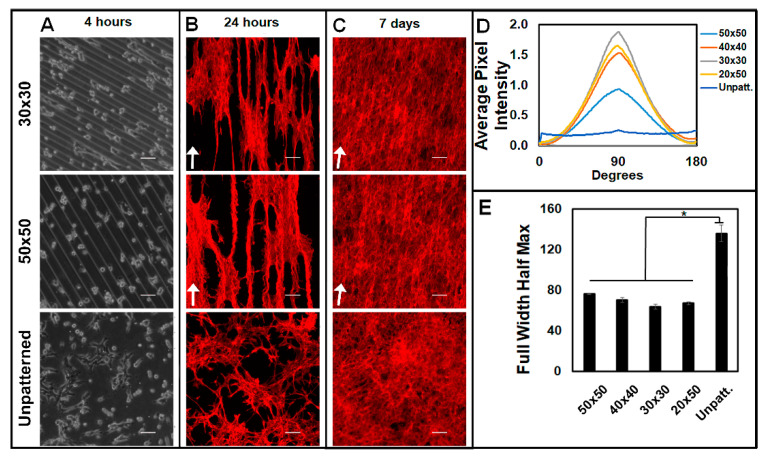
Cell alignment on substrates over 7 days. Cell morphology is shown (**A**) after 4 h by phase contrast and (**B**) 24 h and (**C**) 7 days by visualization with rhodamine phalloidin. (**A**–**C**) Scale bars = 100 µm. (**D**) Radial sum curves for alignment of confluent cell cultures with 10 µm heights after 7 days. (**E**) Full width half maximum values obtained from radial sum curves n = 5 unique images per substrate condition. Data are presented as mean ± SE, where * *p* ≤ 0.05.

**Figure 4:**
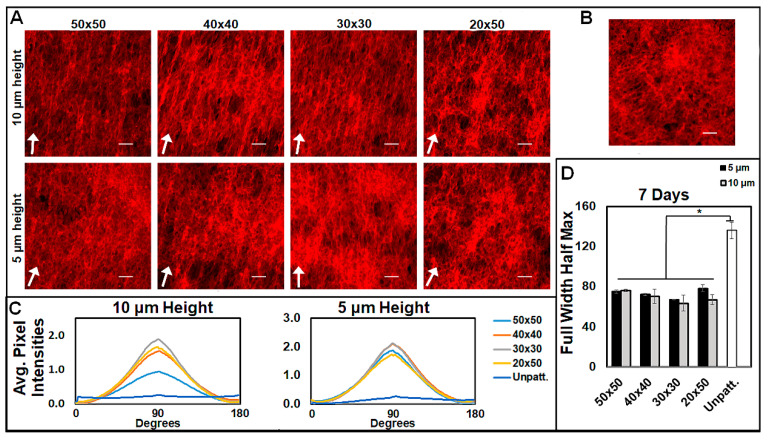
Cell alignment following 7 days of culture compared by pattern height. Cells were cultured on (**A**) patterned and (**B**) unpatterned PDMS for 7 days and visualized with rhodamine phalloidin, with white arrows indicating pattern directions. Scale bar = 100 µm. (**C**) Fast Fourier transform (FFT) analysis quantified the alignment of confluent cell culture substrates after 7 days for each pattern height. (**D**) Full width half maximums derived from average pixel intensity curves (**C**) for both 5 and 10 µm. (**C**,**D**) 10 µm-height full width half maximums reproduced from Figure 3D,E, n = 3 unique images per condition. Data are presented as mean ± SE, where * *p* ≤ 0.05.

Cells were then analyzed over multiple time intervals to examine the prolonged alignment of cells due to the physical constraints of the micropatterns. Changes in cell behavior were evident as early as 4 h of culture (Figure 3A). After 24 h of culture, the cells still adhered primarily to the troughs of the micropatterns for both 10 and 5 μm heights (Figure 3B). The micropatterns promoted the alignment of cells for all the geometries tested (Figure 3B and Figure S2A,B). An FFT analysis performed at 24 h showed significantly more cellular alignment on micropatterned surfaces than on unaligned surfaces at both patterned heights, with similar degrees of alignment across the different micropatterned dimensions (Figure S2C,D). At 7 days, confluent cells were aligned along the pattern direction in comparison to unpatterned cells (Figure 3C). To quantify the alignment of the confluent cells, FFTs were performed, showing that all the micropattern conditions (50 × 50, 40 × 40, 30 × 30, and 20 × 50 at 5 and 10 μm heights) aligned confluent cells significantly better than unpatterned substrates (Figure 3D,E and Figure 4A–C). Statistically, there was no significant difference in alignment between micropatterns for both the 5 and 10 µm pattern heights (Figure 3E and Figure 4D). Additionally, there were no significant differences in the ability of 5 and 10 um pattern heights to induce alignment (Figure 3E and Figure 4D).

### 3.2. Micropatterns Facilitate Aligned ECM Assembly

**Figure 5:**
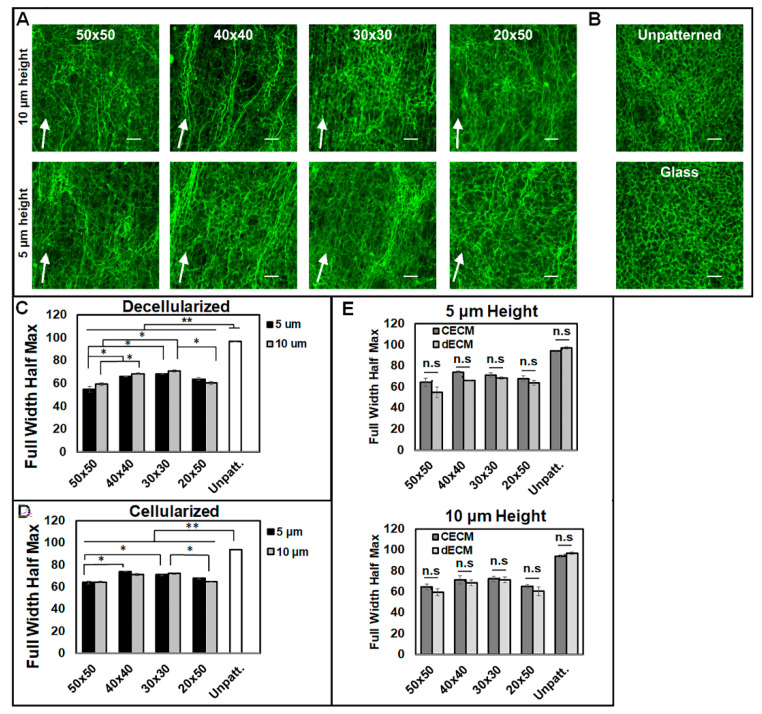
Decellularized, cell-assembled extracellular matrix (ECM) on (**A**) micropatterned PDMS for both pattern heights. (**B**) Decellularized, cell-assembled ECM on unpatterned PDMS (top) and glass coverslip (bottom). White arrows indicate pattern directions. Scale bars = 100 μm. Fibronectin (FN) fibrils visualized with R457 rabbit anti-fibronectin polyclonal antiserum (**C**) Full width half maximum calculations for decellularized matrices. (**D**) Full width half maximum calculations for matrices prior to decellularization. (**E**) Effect of pattern height on the differential alignment of deposited FN matrices for both decellularized and non-decellularized conditions. n = 3 unique images per condition. Data are presented as mean ± SE, where * *p* ≤ 0.05, ** *p* ≤ 0.005.

For the analysis of the alignment of the matrix assembly from aligned cells, fibroblasts were cultured for 7 days, decellularized, and stained for fibronectin. ECM was analyzed before and after decellularization to confirm the alignment of fibrils following the decellularization assay. It was found that the cells cultured on micropatterned surfaces produced an aligned decellularized ECM oriented along the direction of the micropattern grooves (Figure 5A) in comparison to the unaligned matrix on both unpatterned PDMS and cover slips (Figure 5B). FFT analysis confirmed the alignment produced by micropatterned PDMS surfaces following decellularization (Figure 5C) and before decellularization (Figure 5D). Furthermore, the alignment of the decellularized matrices was not significantly different from that of the non-decellularized matrices at either pattern height (Figure 5E). Overall, the micropatterns induced similar trends in alignment for non-decellularized and decellularized matrices. In contrast to the alignment of cells, the decellularized 50 × 50 micropatterns produced a significantly more aligned FN matrix than the 30 × 30 and 40 × 40 patterns for both the 5 and 10 μm height patterns (Figure 5C). Furthermore, the 20 × 50 patterns assembled a significantly more aligned matrix than the 30 × 30 patterns with the 10 μm-height patterns. There was no significant difference found between the heights of the patterns at the same micropatterned dimensions.

### 3.3. Aligned ECM Promotes Guided Cell Adhesion and Migration

**Figure 6:**
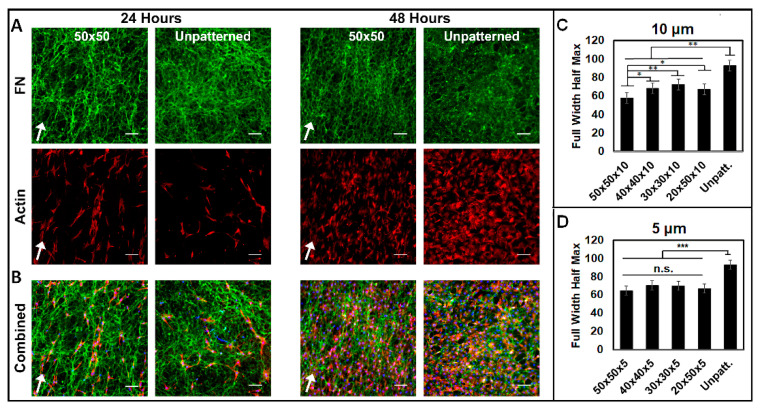
Decellularized ECM (dECM) that was recellularized shows alignment of cells along matrix fibrils. Cells were reseeded on decellularized ECM and cultured for 24 (left) and 48 (right) hours. (**A**) Bioactive ECM surface was stained for fibronectin (green), and cells were visualized with rhodamine phalloidin (red). (**B**) Combined images depicting bioactive ECM surface with fibronectin, actin, and DAPI (blue). White arrows indicate pattern directions. Full width half maximum calculations for cells seeded on decellularized matrices deposited on both (**C**) 10 and (**D**) 5 µm biomaterial pattern heights. n = 3 unique images per condition. Data are presented as mean ± SE. * *p* ≤ 0.05, ** *p* ≤ 0.01, *** *p* ≤ 0.005.

To determine the ability of the aligned dECMs to control cell function, fibroblast cells were seeded onto decellularized matrices and analyzed for adhesion, motility, proliferation, and alignment. The cells were analyzed on dECMs at 24 and 48 h to evaluate single-cell morphology (Figure 6A,B). The cells readily adhered to both patterned and unpatterned decellularized ECMs. The cells homogenously adhered on the aligned dECM, showing a clear preference for the matrix but no observable preference for the underlying PDMS troughs or ridges. FFT analysis showed that after 24 h, the cells seeded on aligned dECMs were significantly more aligned than those on the unaligned dECMs at both patterned heights (Figure 6C,D). Interestingly, for the 10 μm pattern heights, the cells seeded on the dECMs of the 50 × 50 patterns were significantly more aligned than those seeded on the dECMs of the 30 × 30, 40 × 40, and 20 × 50 patterns (Figure 6C). By contrast, there was no significant difference in the alignments of cells on the dECMs assembled on 5 μm-height patterns (Figure 6D).

**Figure 7:**
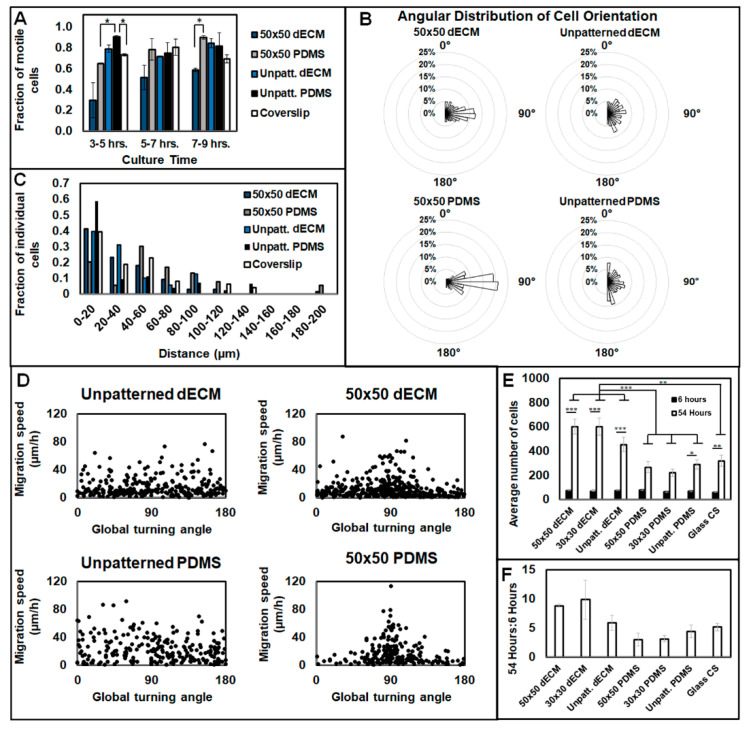
Migration, motility, and proliferation of cells seeded on decellularized ECM. (**A**) The fractions of motile cells for each surface type displayed across two-hour cell culture increments. (**B**) Angular distribution of cell orientation displays the direction of cell migration in 1-h time step increments. Angular bins (10°) illustrate the distribution of orientation across trials. (**C**) Fraction of cells displaced across 3 to 8 h of culture for each unique surface. (**D**) Stepwise cell migration speeds as a function of the global turning angle, with respect to the micropattern (where applicable). (**A**–**D**) Cells were seeded at a density of 5000 cells cm^−1^ and cultured for 3 h before time-lapse imaging. n = 2 unique trials per condition with (**A**) >75, (**B**,**D**) > 210, or (**C**) >20 cells. (**E**,**F**) Proliferation of fibroblast cells on decellularized matrices. (E) Total cells present per image after 6 and 54 h. (**F**) Ratio of the cell counts after 54 h to the average cell counts after six hours. (**E**,**F**) Cells were seeded at a density of 30,000 cells cm^−1^ on both patterned and unpatterned dECM and PDMS; n = 5 images for each unique trial, with 3 unique trials per condition. Data are presented as mean ± SE, where * *p* ≤ 0.05, ** *p* ≤ 0.005, and *** *p* ≤ 0.0005.

To observe the control of cell migration by dECM, time-lapse microscopy was performed following three hours of initial cell adhesion, and cells were tracked for 10 h. At all points post-seeding, the 50 × 50 dECM patterns showed significantly fewer motile cells than the 50 × 50 PDMS, unpatterned dECM, unpatterned PDMS, and standard coverslips (Figure 7A). Both of the 50 × 50 patterned substrates (dECM and uncoated PDMS) induced cell migration parallel to the pattern direction, compared to all the unpatterned conditions, with random migratory patterns (Figure 7B). However, the directionality was less concentrated on the 50 × 50 dECMs compared to that on the 50 × 50 PDMS substrates, where cells also migrated longer distances (Figure 7C). Cells were shown to migrate the quickest on the 50 × 50 patterns (both dECM and PDMS) at parallel orientations to the pattern directions; however, there were no prevailing differences in cell velocity for all the conditions (Figure 7D). 

To analyze the bioactivity of the dECM regarding proliferation, cell numbers were tracked over time. After 54 h, there was significantly more proliferation on the dECM substrates than the PDMS and glass coverslips (Figure 7E). Furthermore, the ratio of cells at 54 h to those at 6 h was notably higher for the dECM substrates than other conditions, but the effect was not significant (Figure 7F). At 54 h, individual differences were not present between the micropatterns, where the 50 × 50 and 30 × 30 patterns did not incur a significantly different effect on proliferation for either the dECM substrates or PDMS substrates (Figure 7E).

## 4. Discussion

In this work, substrates were micropatterned to physically guide cell alignment, elongation, and orientation to ultimately control the spatial orientation of the resulting cell-assembled ECM, as it is established that the secreted ECM is directly related to the contractibility and conformation of cells [18]. While cells predominantly adhered to the troughs of the micropatterns initially, they spread and aligned across the 3D PDMS surfaces and grew to confluence with both the 5 and 10 μm patterned heights. Micropatterned topographies have been used to control the morphology and phenotypes of a multitude of cells previously, including fibroblasts, embryonic stem cells, macrophages, endothelial cells, and epithelial cells [36,37], yet little work has progressed to analyzing the resulting matrix assembly. Here, we show the ability of biomaterials to control cell behavior and subsequent matrix deposition by utilizing strictly 3D, micron-dimension physical cues. Using this, aligned dECM was successfully achieved and showed unique potential for the guidance of cell function as a biomaterial, as reseeded fibroblast cells displayed similar directionality in adherence, motility, alignment, and elongation on dECM substrates as compared to those on standard micropatterned PDMS surfaces.

Consistent with previous work, smaller geometries that physically constricted the cells produced smaller cell areas and a higher degree of alignment [38]. All the micropatterned conditions resulted in more elongation (with smaller areas) than the flat surfaces, thereby negating concerns that larger pattern dimensions may act as a flat surface [39]. This effect was likely augmented by our increased depth of structures compared to that of many other topographies analyzed, which mitigated the effect of the ridge size, as the cells were able to spread in troughs and elongate [40]. Furthermore, the cells utilized in our study had an average diameter of 18 µm, thus extenuating the ability of the smaller patterns to physically compress the cytoskeleton. The effect of ridge width was also prevalent in alignment analysis, with larger patterned dimensions resulting in less-aligned cells after 24 h and at confluency. Furthermore, pattern depths also showed a measure of control on alignment, with more alignment displayed on 10 µm-height patterns than 5 µm-height patterns at confluency. Dunn and Brown demonstrated that ridge width is more important than groove width in controlling alignment and elongation, observing that focal contacts and actin bundles are primarily localized on ridges themselves and rarely experience growth between each localization. They subsequently postulated an inverse relationship between elongation and alignment relative to ridge size [41]. To further augment these claims, it was notable that cells on 20 × 50 patterns, with an identical ridge size to the 50 × 50 pattern but smaller groove widths, were less elongated and less aligned than the cells on the 30 × 30 and 40 × 40 patterns, though similar to the cells on the 50 × 50 patterns. A reasonable inference is that 2D pattern geometry (ridge and groove size) was dominant in mediating the morphological trends, while our relatively large pattern depths of 5 and 10 µm extenuated these effects. 

Towards a solution for tissue engineering, the ability to fabricate aligned cells along micropatterned grooves presents a unique ability to take a step further and control the resulting ECM alignment for a variety of different strategies. It has been previously demonstrated that cell morphology is an ample indicator of resultant ECM morphology, and as cells adhere and align on micropatterns, cell-secreted proteins assemble to form a complex, organized matrix through protein–protein and cell–protein interactions [11,13,40]. FN is a prominent ECM assembly protein and is responsible for forming a provisional matrix, which is mediated by cytoskeletal tension [42]. Following decellularization, FN fibrils were significantly more aligned on micropatterned PDMS surfaces than flat PDMS and standard coverslips, with the FN alignment staying consistent before and after decellularization. An interesting observation between the cells on the micropatterned surfaces and the alignment of the resultant ECM was that, in contrast to the alignment of the FN matrices, individual fibroblasts were significantly more aligned on the 40 × 40 and 30 × 30 patterns than the 50 × 50 and 20 × 50 patterns. Regarding this, smaller pattern geometries (40 × 40 and 30 × 30) may have inhibited the spread of cells and cell-secreted proteins, specifically the FN fibrils, and thereby prevented the assembly of a dense matrix in a similar pattern to the cells. Meanwhile, patterns with wider ridges (50 × 50 and 20 × 50), though less adept at controlling the alignment of individual cells, were large enough to promote adjacent cell-to-cell and cell–protein interactions that are crucial to the formation of an organized, aligned ECM [43]. It is also worthwhile to speculate on the effects of pattern geometry on individual proteins. Tenascins, in particular, can diminish the adhesive capacity of FN, for both cells and other proteins, and thereby respond to mechanical stress by regulating the attachment of the cell to the ECM, which is observed in the upregulation of Tenascin-C during tissue repair [44]. From a biomolecular perspective, the increased surface contact between cells and the micropattern geometry may increase anti-adhesive signals for the 40 × 40 and 30 × 30 patterns, and thus hinder cell–FN interactions, which are known to promote alignment [45].

The production of dECM on micropatterned surfaces also better emulates the geometrical organization of native, fibrillar matrices found in regenerative microenvironments. By contrast, healthy tissue matrices—where integrins anchor cells to the connective matrix or, in many cases, basement membranes—typically exhibit lower elastic strength and more variable geometry [46,47,48]. Thus, while the induced alignment of dECMs offers better control over cell behavior, it simultaneously mimics a proliferative environment for the repair of injured tissue. Fibroblasts seeded on 30 × 30 and 40 × 40 dECM substrates were, again, significantly more aligned than those on 50 × 50 and 20 × 50 patterns, following an identical trend to that of the previously described ECM substrate alignment. This effect was more pronounced with 10 µm-height patterns than 5 µm-height patterns, though it has been shown that sufficient control of fibroblasts can be attained at much smaller depths and areas, as reflected by the multitude of work using nanopatterned surfaces [49]. The cell cytoskeleton is remodeled by communication with ECM cues through focal adhesions, which, in turn, mediates cell responses such as alignment. For a regenerative microenvironment, it is expected that cells would likewise align, elongate, and proliferate in conjunction with the preexisting ECM topography [50]. However, in analyzing migratory capacity, significantly fewer cells were motile on patterned 50 × 50 dECM substrates than on unpatterned dECM. Furthermore, significantly fewer cells under both dECM conditions (whether patterned or unpatterned) were motile than their respective PDMS counterparts. This is easily attributable to the increased focal adhesion sites on the dECMs relative to those on the PDMS substrates alone. However, it was not exhibited in how the ECM physical properties differed from those on the patterned substrates. For instance, matrix stiffness is an important factor in the motility of cells in development, regeneration, and cancer [47]. However, while the stiffness of PDMS substrates can be manipulated to affect cell migration [49], it is notably more difficult to utilize that effect with complex biological proteins. Regardless, cell migration speed was similar on both dECM substrates and PDMS. Furthermore, and perhaps relevantly in the context of regeneration, proliferation on dECM was significantly more prevalent than that on PDMS or standard coverslips, showing the bioactivity and utility of the dECM surface.

## 5. Conclusions

Micropatterned PDMS substrates were designed to facilitate the deposition and assembly of an aligned ECM from fibroblast cells for use as tissue engineering biomaterials. Fibroblast cells aligned, spread, and assembled matrix along the 3D topography and were decellularized to retain a bioactive, aligned ECM on the material. The geometry of the PDMS micropatterns was optimized for dECM alignment and density to promote the subsequent alignment, spreading, proliferation, and migration of cells cultured on the dECM. This work harnessed the prevalence of fibroblasts and their distinct role in ECM assembly and tissue repair to form a native, bioactive surface for biomaterials. Going forward, this presents a tremendous opportunity for optimizing and incorporating bioactivity into engineered surfaces for a variety of tissue engineering applications.

## Supplementary Materials

The following are available online at https://www.mdpi.com/2306-5354/7/3/102/s1, Figure S1: Soft lithography protocol for fabrication of micropatterned PDMS biomaterial. The photoresist viscosity and parameters for spin-coating speed, soft bake temperature, photolithography exposure, and post exposure temperature weredependent on the desired three-dimensional pattern height, Figure S2. Cell alignment following 24 h of culture. Cells were cultured on (A) patterned and (B) unpatterned PDMS for 24 h and visualized using rhodamine-phalloidin, wherewhite arrows indicate pattern direction. Scale bar = 100 μm. (C) FFT analysis quantified alignment of 24-h cell culture on substrates. (D) Full width half maximum values derived from average pixel intensity curves. n = 3 unique trials for each condition were performed. Data are presented as mean ± SE, where * *p* ≤ 0.05, ** *p* ≤ 0.005, and *** *p* ≤ 0.0005. Table S1: Ridgeand trough dimensions of PDMS substratesas verified across three unique surfaces, n > 100 measurements per dimension.
