# Supplementary material for: Micropatterning Decellularized ECM as a Bioactive Surface to Guide Cell Alignment, Proliferation, and Migration
Source: Bioengineering (Basel). 2020 Aug 31;7(3):102. doi: 10.3390/bioengineering7030102 (PMC7552701; doi:10.3390/bioengineering7030102)
Supplement: Supplementary file 1 [file bioengineering-07-00102-s001.pdf]

Supplemental Figures

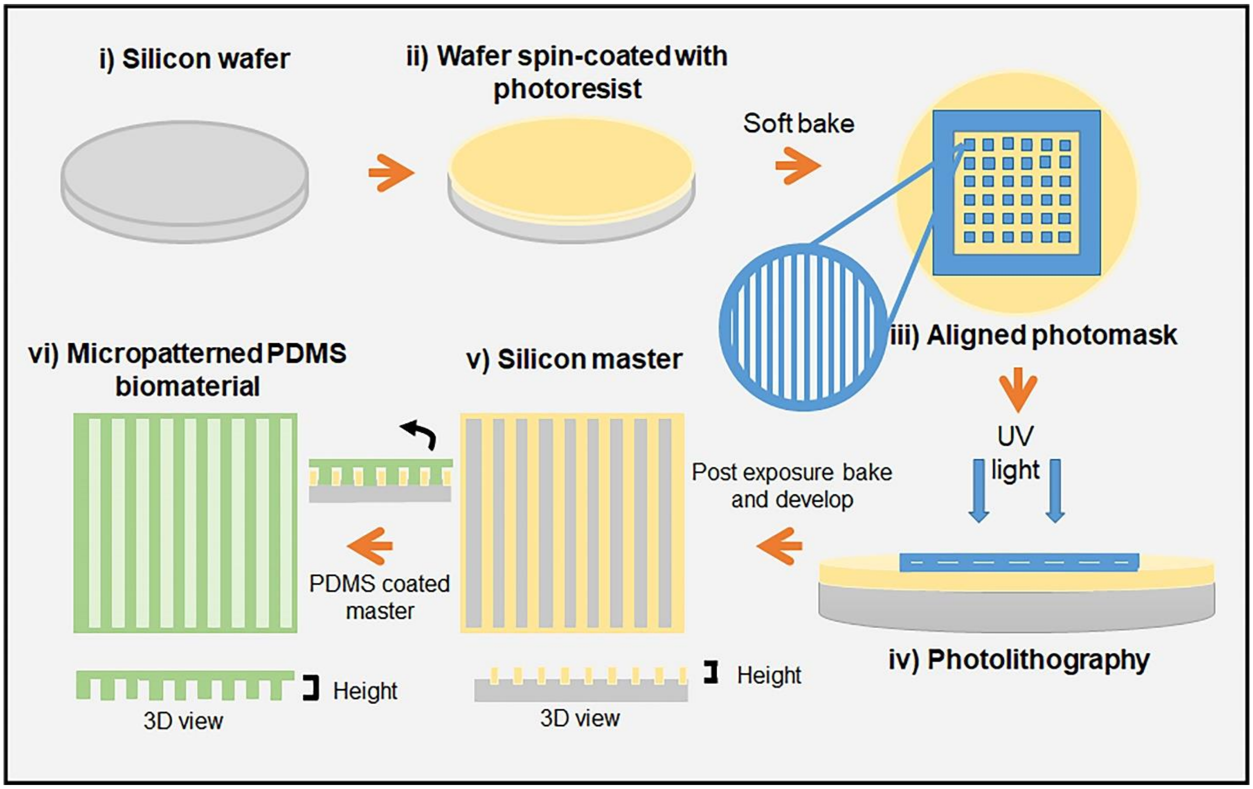

Figure S1. Soft lithography protocol for fabrication of micropatterned PDMS biomaterial. The photoresist viscosity and parameters for spin-coating speed, soft bake temperature, photolithography exposure, and post exposure temperature were dependent on the desired three-dimensional pattern height.

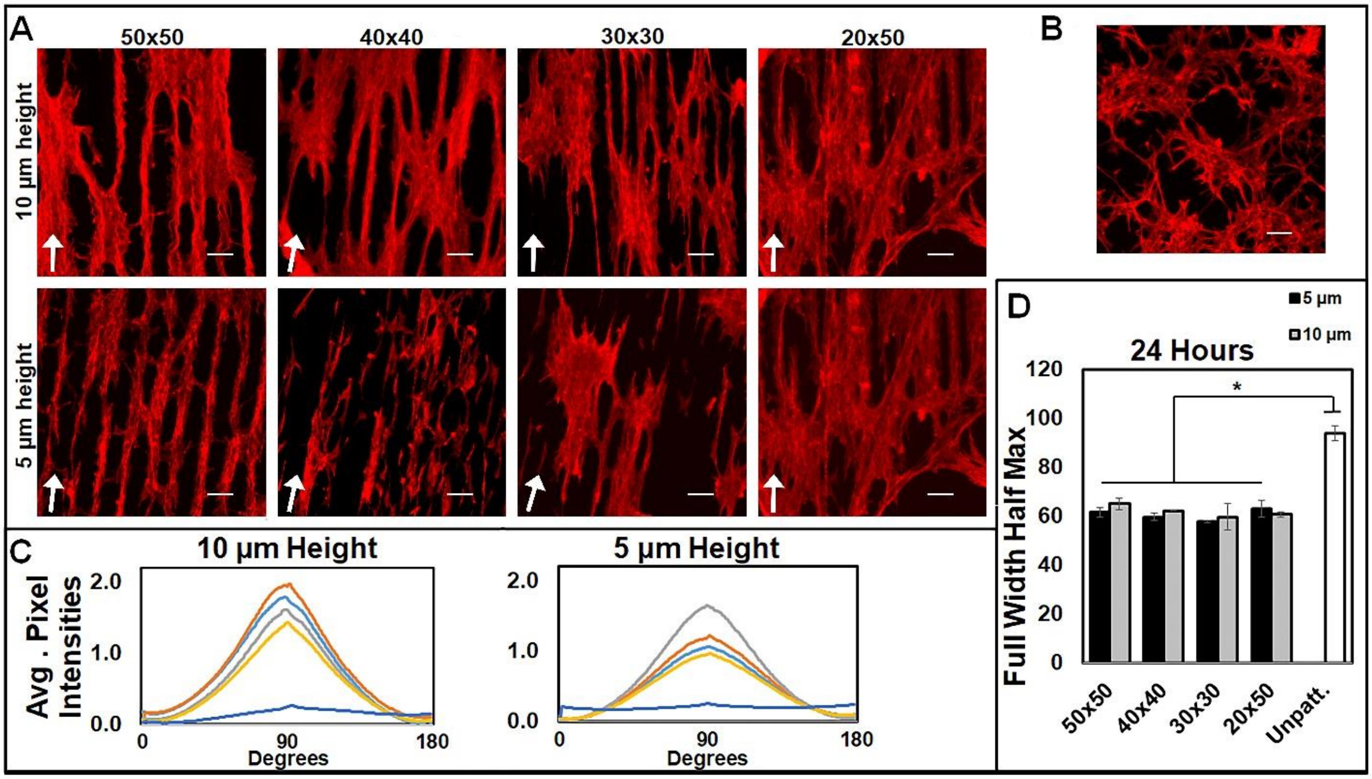

Figure S2. Cell alignment following 24 hours of culture. Cells were cultured on (A) patterned and (B) unpatterned PDMS for 24 hours and visualized using rhodamine-phalloidin, where white arrows indicate pattern direction. Scale bar = 100 µm. (C) FFT analysis quantified alignment of 24-hour cell culture on substrates. (D) Full width half maximum values derived from average pixel intensity curves. n = 3 unique trials for each condition were performed. Data are presented as mean ± SE, where * ≤ p 0.05, **p ≤ 0.005, and ***p ≤ 0.0005.

Dimensions	10 µm	5 µm
Height	10.3 ± 0.1	5.3 ± 0.2
50x50 Ridge	50.3 ± 0.9	49.9 ± 0.4
50x50 Trough	50.8 ± 0.9	50.4 ± 0.7
40x40 Ridge	39.2 ± 0.8	40.3 ± 0.3
40x40 Trough	41.9 ± 0.6	41. ± 1
30x30 Ridge	30.4 ± 0.7	29.2 ± 0.2
30x30 Trough	30.5 ± 0.7	31.2 ± 0.3
20x50 Ridge	21.6 ± 0.4	20.7 ± 0.3
20x50 Trough	50.9 ± 0.8	49. ± 2

Table S1. Ridge and trough dimensions of PDMS substrates as verified across three unique surfaces, n > 100 measurements per dimension.
